# A different lens to explore health literacy skills in atopic conditions

**DOI:** 10.1016/j.jacig.2024.100368

**Published:** 2024-11-12

**Authors:** Deryn Thompson

**Affiliations:** UniSA Clinical and Health Sciences, University of South Australia, Adelaide South, Australia

To the Editor:

The commendable work of Leeman and Loman demonstrated associations between low health literacy (HL) in patients with atopic dermatitis (AD) in The Netherlands, impaired health-related quality of life, and older age.^1^ Their finding is important, but I write this letter to highlight the fact that researchers in HL regarding AD and allergic conditions continue to overlook the significance of the learning process and cognitive learning principles in HL skills development and empowerment.^1^, ^2^, ^3^ Like other researchers examining HL in the area of allergy,^3^ Leeman and Loman focus on “information,”^1^ overlooking the fact that cognition and learning are paramount in the active transformation of information to knowledge.^2^ Information becomes knowledge through a learning process, thus influencing patients’ health behaviors.^2^ People can then work toward autonomy and self-management, thereby guiding health outcomes and their use and navigation of health services to manage their AD and allergic conditions.^2^

Researchers and health professionals (HPs), including clinicians and nurses, can consider viewing HL through the lens of cognitive learning principles. In 2022, Thompson et al mapped the cognitive learning principles used by nurses in parent education practice, as well as which principles parents identified as important to their learning experiences. Thompson et al argue that despite educational psychology having determined that cognition is key in the learning process of patient-education and a critical point within the HL journey, no framework existed to guide HPs’ practice.^2^ Educational psychology research underscores that when information is provided to the patient by HPs through clear communications, the learner (patient) uses cognition, takes the new information and links it to what they know already, thinks about it in new ways, reshapes it, and starts to transform it into knowledge that is meaningful to his or her situation.^2^ Information itself is just data, but when it becomes knowledge, learners can then see ways in which they can use their knowledge to manage their health condition, apply reasoning, and make active decisions.^2^^,^^4^ I argue that HPs must recognize the value in helping patients develop these skills for HL and self-manage their health.

Thompson et al collected data by observation, interview, focus groups, and a workshop.^4^ Thematic analysis showed alignment with the Dimensions of Learning framework from educational psychology (Table I [column 1]), which has guided educators for 3 decades, producing students who could think, reason, and problem-solve, following their information to knowledge transformation.^5^

**Table I:** The Dimensions of Learning framework and examples in allergy practice

The 5 Dimensions of Learning^5^	Practical examples of cognitive learning principles assisting in HL for atopic conditions
Attitudes and perceptions: readiness, a positive attitude to learn that is influenced by internal situations (eg, emotions), a safe learning environment, learners seeing value and relevance of the learning and having clearly defined learning goals and motivation	New diagnosis of an incurable but controllable allergic condition (eg, AD, food allergy, angioedema, anaphylaxis), psychosocial or financial stressors? Previous poor experiences with HPs? Conflicting advice, no explanations or demonstrations? Fear of medications and/or treatments or ineffectiveness. What do patients want from education sessions? HPs must listen to the answers
Acquisition and integration of knowledge: understanding strategies for linking new knowledge to existing knowledge; organizing, processing, and storing knowledge for later retrieval and use (declarative knowledge [facts] and procedural [practical] knowledge); shaping and internalizing skills to use later	Identify HL level. *Facts*: basic explanation about the allergic condition: what is happening and why (eg, in cases of AD, skin is dry and itchy, gaps in barrier, brick wall analogy, action of TCSs/flare control creams, why and when to use them, and how much to use. *Anaphylaxis*: triggers, symptoms, action plan (why an autoinjector must be used promptly). Reactions are not the patients’ or family’s “failure.” *Practical knowledge*: demonstrate (in cases of AD, application of emollient or a TCS [show how much to use]; in cases of anaphylaxis, demonstrate autoinjector use), step through the care plan, practice use, refine the use of treatments at a review appointment, and adjust if needed
Extension and refinement of knowledge: strategies for accomplishing this include comparing, classifying, making inductions and deductions, analyzing perspective, correcting errors, applying reasoning, gaining perspectives, and using analogies. These processes help learners deconstruct and reconstruct knowledge, enabling them to think of their new knowledge and skills in new and unusual ways	The patient and family continue to execute the care plan. The HP checks in to see how treatments (application or use) are going at home. For example, the patient hated ointment and/or wet wraps or feared using an autoinjector. The HP works with the patient on solutions (thinking/reasoning), as he or she builds confidence to monitor symptoms and discern skin or allergic changes. Praise helps the patient’s motivation; the patient sees value with outcome: (in the case of AD, positive changes in skin, reversal of anaphylaxis as a result of autoinjector use), less fear, fewer symptoms
Application of the knowledge meaningfully: strategies for accomplishing this include decision making, problem solving, and analysis	Fine-tune skills, the patient persists with treatments, can adapt if symptoms recur, recognizes the triggers (eg, when the patient’s illness [AD] flares or food eaten leads to anaphylaxis). Praise from the HP as the patient perseveres, develops resilience, and understands. The patient starts to see value in lifestyle and behavior changes, can discern and reject non–evidence-based misinformation. The HP provides basic scenarios; the patient answers with what he or she will do. Reflects the patient’s thinking, reasoning, problem-solving skills, as well as confidence to persevere with a lifelong condition, but manage effectively. The patient can explain to others what he or she is doing and why, use reshaped knowledge, and make decisions
Habits of mind: thinking and learned skills become “part of everyday life”; learners master critical, creative, self-regulated thinking and operate autonomously. The patient and family develop a sense of agency in management of their health condition	Self-management/efficacy becomes second nature. The patient incorporates the lifestyle changes needed, understands his or her condition (triggers and treatments), and the need for early intervention (if AD flares or if anaphylaxis occurs). The patient feels confident and capable of avoiding triggers, and initiating and managing adaption of treatments; he or she knows and states the “why, how, what, and when” of ongoing evidence-based care. The patient is now a good critical thinker!

*TCS*, Topical corticosteroid.

I argue that using such a framework can shed new light on HL and self-management and efficacy, which require patients and carers to develop the skills to think, reason, and problem-solve about their fluctuating health conditions, such as AD and allergies. Without these skills, even straightforward information is hard to use, especially to make informed decisions, which is a goal of HPs for patients and carers.^4^ Using the framework’s 5 dimensions enables HPs to reflect on their current practice; see links to cognitive learning principles that they may be using (for examples, see Table I [column 2]); and recognize the importance of the information transformation process in effective learning, HL journeys, self-care, and behavior changes. Researchers must further explore links between using the Dimensions of Learning framework, integrating cognitive learning principles into patient education, patients’ HL journeys, and learning outcomes.^4^^,^^5^

Ethical approval for the PhD research on which this correspondence is based was granted by the Human Research Ethics committees of the Women’s and Children’s Health Network (approval no. HREC/17/WCHN/41) and the University of South Australia (approval no. 200009) and Women’s and Children’s Health Network Research Governance body (approval no. SSA/17WCH/045).

## Disclosure statement

Disclosure of potential conflict of interest: D. 10.13039/100004686Thompson reports relationships with Eczema Support Australia and Quality Use of Medicines 10.13039/100027925Alliance that include consulting or advisory activity, as well as speaking and lecture honorarium for relationships with L’Oréal France SNC and Ego Pharmaceuticals Pty, Ltd.
